# Fragilidade em pessoas idosas com Diabetes Mellitus e fatores associados: estudo longitudinal*

**DOI:** 10.15649/cuidarte.3191

**Published:** 2023-12-25

**Authors:** Rosalina Aparecida Partezani Rodrigues, Jakeline Silva de Araújo, Jéssica Silva de Araújo, Daiane de Souza Fernandes, Mauriely Paiva de Alcantara e Silva, Fernanda de Brito Matiello, Vanderlei José Haas, Jack Roberto Silva Fhon

**Affiliations:** 1 . Universidade de Sáo Paulo, Ribeiráo Preto, Brasil. Email: rosalina@eerp.usp.br Universidade de São Paulo Universidade de Sáo Paulo Ribeiráo Preto Brazil rosalina@eerp.usp.br; 2 . Universidade de Sáo Paulo, Ribeiráo Preto, Brasil. Email: jaki-araujo@outlook.com Universidade de São Paulo Universidade de Sáo Paulo Ribeiráo Preto Brazil jaki-araujo@outlook.com; 3 . Universidade de Sáo Paulo, Ribeiráo Preto, Brasil. Email: jeesaraujoo@gmail.com Universidade de São Paulo Universidade de Sáo Paulo Ribeiráo Preto Brazil jeesaraujoo@gmail.com; 4 . Universidade de Sáo Paulo, Ribeiráo Preto, Brasil. Email: daissf@usp.br Universidade de São Paulo Universidade de Sáo Paulo Ribeiráo Preto Brazil daissf@usp.br; 5 . Universidade de Sáo Paulo, Ribeiráo Preto, Brasil. Email: maurielypaiva@usp.br Universidade de São Paulo Universidade de Sáo Paulo Ribeiráo Preto Brazil maurielypaiva@usp.br; 6 . Universidade de Sáo Paulo, Ribeiráo Preto, Brasil. Email: fernanda.matiello@usp.br Universidade de São Paulo Universidade de Sáo Paulo Ribeiráo Preto Brazil fernanda.matiello@usp.br; 7 . Universidade Federal do Triangulo Mineiro, Uberaba, Brasil. Email: vjhaas@uol.com.br Universidade Federal do Triângulo Mineiro Universidade Federal do Triangulo Mineiro Uberaba Brazil vjhaas@uol.com.br; 8 . Universidade de Sáo Paulo, Sáo Paulo, Brasil. Email: betofhon@usp.br Universidade de São Paulo Universidade de Sáo Paulo Sáo Paulo Brazil betofhon@usp.br

**Keywords:** Idoso, Diabetes Mellitus, Fragilidade, Fatores de risco, Aged, Diabetes Mellitus, Frailty, Risk Factors, Anciano, Diabetes Mellitus, Fragilidad, Factores de Riesgo

## Abstract

**Introdujo:**

O Diabetes Mellitus ocasiona a diminuido das reservas e capacidades funcionais. Sua associacáo com a síndrome da fragilidade acarreta declínio gradativo no sistema biológico, causando prejuízos globais a saúde da populado idosa e, portanto, comprometendo sua qualidade de vida.

**Objetivo:**

analisar a evolucáo da fragilidade e fatores associados em pessoas idosas com Diabetes Mellitus.

**Materiais e Métodos:**

Estudo longitudinal caracterizado por duas avaliacóes com intervalo de 18 meses, envolvendo 49 participantes com idade > 60 anos e de ambos os sexos com diagnóstico clínico de Diabetes Mellitus. Na análise dos dados foram utilizadas medidas de tendencia central, dispersáo, proporcóes, teste náo paramétrico de Wilcoxon pareado e análise linear múltipla.

**Resultados:**

Na avaliacáo do seguimento, ocorreu um aumento da fragilidade e comprometimento da capacidade funcional entre as duas avaliacóes. Fatores associados, como as atividades instrumentais da vida diária e o número total de doencas, impactaram negativamente sobre a fragilidade dos participantes.

**Discussáo:**

Os resultados encontrados no estudo convergem com a literatura cientifica relacionada a associacáo de doencas crónicas como a Diabetes Mellitus no aumento da fragilidade.

**Conclusáo:**

A presenca de uma doenca crónica como a Diabetes Mellitus pode ocasionar o aumento da fragilidade e comprometer a funcionalidade. A avaliacáo destas condicóes nos servicos de saúde para identificacáo precoce é fundamental para estabelecer estratégias assertivas para a manutencáo de um envelhecimento com qualidade de vida.

## Abstract (en)

**Introduction:**

Diabetes Mellitus causes a decrease in reserves and functional capabilities. Its association with frailty syndrome leads to a gradual decline in the biological system, causing global harm to the health of the elderly population and, therefore, compromising their quality of life.

**Objective:**

To analyze the evolution of frailty and associated factors in elderly people with Diabetes Mellitus.

**Materials and Methods:**

Longitudinal study characterized by two assessments 18 months apart, involving 49 participants aged > 60 years and of both sexes with a clinical diagnosis of Diabetes Mellitus. In data analysis, measures of central tendency, dispersion, proportions, paired Wilcoxon non-parametric test and multiple linear analysis were used.

**Results:**

In the follow-up assessment, there was an increase in frailty and impairment of functional capacity between the two assessments. Associated factors, such as instrumental activities of daily living and the total number of illnesses, had a negative impact on the participants' frailty.

**Discussion:**

The results found in the study converge with the scientific literature related to the association of chronic diseases such as Diabetes Mellitus with increased frailty.

**Conclusion:**

The presence of a chronic disease such as Diabetes Mellitus can cause increased frailty and compromise functionality. The assessment of these conditions in health services for early identification is essential to establish assertive strategies for maintaining aging with quality of life.

## Resumen

**Introducción:**

La Diabetes Mellitus provoca una disminución de las reservas y capacidades funcionales. Su asociación con el síndrome de fragilidad conduce a un paulatino deterioro del sistema biológico, provocando un daño global a la salud de la población anciana y, por tanto, comprometiendo su calidad de vida.

**Objetivo:**

Analizar la evolución de la fragilidad y factores asociados en personas mayores con Diabetes Mellitus.

**Materiales y Métodos:**

Estudio longitudinal caracterizado por dos evaluaciones con 18 meses de diferencia, involucrando a 49 participantes con edad > 60 años y de ambos sexos con diagnóstico clínico de Diabetes Mellitus. En el análisis de los datos se utilizaron medidas de tendencia central, dispersión, proporciones, prueba no paramétrica de Wilcoxon pareada y análisis lineal múltiple.

**Resultados:**

En la evaluación de seguimiento, hubo un aumento de la fragilidad y el deterioro de la capacidad funcional entre las dos evaluaciones. Los factores asociados, como las actividades instrumentales de la vida diaria y el número total de enfermedades, tuvieron un impacto negativo en la fragilidad de los participantes.

**Discusión:**

Los resultados encontrados en el estudio convergen con la literatura científica relacionada con la asociación de enfermedades crónicas como la Diabetes Mellitus con mayor fragilidad.

**Conclusión:**

La presencia de una enfermedad crónica como la Diabetes Mellitus puede provocar aumento de fragilidad y comprometer la funcionalidad. La evaluación de estas condiciones en los servicios de salud para su identificación temprana es fundamental para establecer estrategias asertivas para mantener el envejecimiento con calidad de vida.

## Introdujo

O Diabetes Mellitus (DM) é uma doenga crónica na qual o corpo nao produz ou nao utiliza adequadamente o hormónio insulina. Possui como principais complicates a cetoacidose e a neuropatia diabética, além de possuir uma alta taxa de morbimortalidade se nao tratada adequadamente e se associada aos fatores de risco como doengas, idade elevada, obesidade, dentre outros^1^.

Estudos apontaram um aumento da incidencia do DM nos últimos anos, com 537 milhóes de pessoas diabéticas no mundo. No Brasil, as estimativas mais recentes preveem 16,8 milhóes de pessoas seráo acometidas da doenga, cerca de 7% da populagáo idosa^2^. Associado a esta situagáo, estima-se que, em 2031, o número da populagáo idosa no Brasil ultrapasse o de jovens, com 102,3 pessoas idosas para cada 100 jovens, o que também tende a tornar-se recorrente o aumento de casos de DM, visto que é uma das doengas mais prevalentes nessa populagáo^3^.

Considerando a mudanza da pirámide etária mundial, o aumento da populagáo idosa com Diabetes Mellitus e fragilidade tem sido cada vez mais reconhecido como problemas de saúde pública, além de impactar diretamente na qualidade de vida e caracterizar desafios para todas as sociedades, principalmente, para países em desenvolvimento como o Brasil^4^.

A fragilidade é caracterizada como uma síndrome que ocasiona a diminuido da resistencia e reserva aos estressores. Tende a proporcionar um estado de vulnerabilidade, devido principalmente ao elevado número de fatores associados e sua alta prevalencia^5^. Estudo de revisáo sistemática mostrou que a prevalencia da fragilidade em pessoas idosas com Diabetes Mellitus foi de 10% a 25%, e esta associagáo impacta no risco de incapacidades, hospitalizares, mortalidade e baixa qualidade de vida, além de provocar o desenvolvimento e progressáo da fragilidade^6^.

Convém ressaltar que a perda de peso, dimensáo do fenótipo físico da fragilidade, vem sendo associada a síndrome da fragilidade, pois alguns medicamentos para o DM, os inibidores do transportador da glicose de sódio, inibidores da alfa-1-glicose, metformina, agonistas do peptídeo1 semelhante ao glucagon podem causar maior perda de peso, aumentando o risco de fragilidade^7^. Além disso, a resistencia a insulina ou a sua deplegáo pode ser um fator importante na progressáo da fragilidade em pacientes com diabetes, uma vez que a insulina é bem conhecida por ser um hormónio anabólico no músculo^8^.

Nesse contexto, a partir do preceito de que a fragilidade pode ocasionar a diminuido da capacidade funcional, a qual pode ser agravada por complicares micro e macrovasculares decorrentes do DM^5^, verifica-se que os fatores de risco associados as condigóes de fragilidade e DM possuem uma relagáo intrínseca com o comprometimento da funcionalidade na pessoa idosa^1^.

Portanto, a associagáo entre Diabetes Mellitus e a fragilidade deve ser investigada devido as possíveis complicagóes para a vida desta populagáo, como, por exemplo, a diminuigáo progressiva da capacidade funcional, internagóes recorrentes e, consequentemente, maior necessidade de utilizagáo dos servigos de saúde em todos seus níveis de atengáo, com destaque para a Atengáo Primária a Saúde, por se tratar da porta de entrada da pessoa idosa no sistema de saúde, possibilitando o reconhecimento da complexidade das suas necessidades e fornecendo uma abordagem abrangente, para melhorar a qualidade de vida e a autonomia^4^. Destaca-se, ainda, a necessidade de pesquisas sobre a temática na populagáo idosa brasileira em estudos de seguimento.

O presente estudo justifica-se pela necessidade de identificado de condigóes que, aliadas ao processo de fragilizagao, podem afetar o envelhecimento saudável da populagao idosa com Diabetes Mellitus, e assim propor direcionamentos nas estratégias de manejo, proporcionando um cuidado integral para melhorar a qualidade de vida. O estudo tem por objetivo analisar a evolugao da fragilidade e fatores associados em pessoas idosas com Diabetes Mellitus.

## Materiais e Métodos

Pesquisa de abordagem quantitativa, observacional e longitudinal por um período de seguimento de 18 meses. A primeira avaliagao (A1) ocorreu presencialmente entre novembro de 2019 a fevereiro de 2020, e a segunda avaliagao (A2), no segundo semestre de 2021, por ligagao telefónica devido as restrigóes ocasionadas pela existencia da pandemia da Covid-19. Em ambas as avaliagóes, as perguntas foram verbalizadas pelo entrevistador, e o tempo média de entrevista foi de 40 minutos por participante. Na A1, a abordagem foi realizada em um consultório do local da coleta, garantindo a privacidade do participante. Na A2, a interpelagao foi conduzida por ligagao telefónica, sendo que, previamente, foi agendado um horário para ratificar o objetivo e duragao da entrevista.

O estudo foi realizado em uma Unidade de Saúde de um município paulista, sendo conduzido conforme as diretrizes Strengthening the Reporting of Observational Studies in Epidemiology (STROBE)^9^.

Os critérios de inclusao para o estudo foram: idade > 60 anos, diagnóstico clínico de Diabetes Mellitus registrado no prontuário do participante, ambos os sexos e na segunda avaliagao, ter participado da primeira etapa do estudo. Para o procedimento de amostragem, considerou-se o total de atendimentos diários de pessoas idosas no local da coleta, que correspondia entre 10 a 12 consultas. Utilizou-se o aplicativo Power Analysis and Sample Size (PASS), v. 15, obteve-se uma amostra mínima de n = 206 participantes para o projeto principal, sendo que destes, 84 (40,80%) apresentaram o diagnóstico médico de Diabetes Mellitus e compuseram a amostra na primeira avaliagao (A1). Na segunda avaliagao (A2) foram entrevistadas 49 pessoas idosas. Ocorreu uma perda amostral de 35 participantes (41,60%) entre as duas avaliagóes, sendo que destes, 23,80% nao foram localizados por contato telefónico após tres tentativas em dias alternados, e 17,80% recusaram-se a participar, compondo a amostra final de 49 participantes.

A variável dependente do estudo foi a fragilidade. Para o seu rastreio, foi utilizada a escala de Tilburg Frailty, a qual avalia a vulnerabilidade e fragilidade da pessoa idosa. Foi revisada e validada no Brasil. Possui 15 questóes objetivas relacionadas aos aspectos físicos (perda de peso, dificuldade para caminhar e manter o equilíbrio, visao e audigao prejudicada e cansago físico), social (suporte social) e psicológico (déficit cognitivo, sintomas depressivos, ansiedade e enfrentamento de problemas). O escore final > 5 mostra resultado indicativo de fragilidade^10^.

As variáveis independentes utilizadas nas análises foram: sexo, estado civil, mora sozinho, idade, escolaridade, renda e número total de doengas. As variáveis sexo, idade e escolaridade, por serem consideradas fatores de confusao, foram mantidas no modelo, independentemente de sua significancia estatística. Para avaliagao das Atividades Instrumentais da Vida Diária (AIVD) foi utilizado o Índice de Lawton e Brody (1969), que engloba atividades sociais mais complexas avaliando a capacidade de o indivíduo conviver em comunidade. A adaptagao e confiabilidade do instrumento que mensura as Atividades Instrumentais da Vida Diária (AIVD) foram realizadas no contexto brasileiro. Possui uma pontuagáo que varia de sete (maior nivel de dependencia) a vinte e um pontos (independencia completa), e a pessoa idosa pode ser categorizada em: dependencia total (7 pontos); dependencia parcial (8 - 20) e independencia (21 pontos), capaz de realizar todas as AIVD sem ajuda^11^.

Para avaliagáo das Atividades Básicas da Vida Diária (AVD), foi utilizado o Índice de Barthel, traduzido e validado no Brasil^12^, que avalia o desempenho para as atividades de alimentado, banho, vestuário, higiene pessoal, eliminado intestinal e vesical, uso do vaso sanitário, transferencia cadeira-cama, deambulado e subir e descer escadas. A pontuagáo varia de zero a 100, sendo que maior pontuagáo indica maior independencia^13^.

Para a análise dos dados foram utilizados os programas estatísticos Statistical Package for the Social Sciences - SPSS v. 22.0 e o programa R (R Core Team, 2022)^14^, versáo 4.1.1. Foram realizadas medidas de tendencia central (média e mediana) e de dispersáo (desvio - padráo) para as variáveis quantitativas e proporgóes para variáveis qualitativas, e o teste náo paramétrico de Wilcoxon pareado na avaliagáo da fragilidade, AIVD e AVD.

Para a análise de regressáo da fragilidade com fatores associados foi realizada a regressáo linear múltipla, sendo previamente feito um levantamento na literatura cientifica das variáveis que apresentavam relagáo com a variável dependente, além do critério da análise bivariada. Para todos os testes estatísticos, a significancia foi p < 0,05.

Os dados da pesquisa estáo armazenados no acervo do Programa Excel do Núcleo de Pesquisa em Geriatria e Gerontologia (NUPEGG) da Escola de Enfermagem de Ribeiráo Preto da Universidade de Sáo Paulo, e no Mendeley Data^15^.

O estudo foi aprovado pelo Comite de Ética em Pesquisa Escola de Enfermagem de Ribeiráo Preto da Universidade de Sáo Paulo (4.206.554, de 11 de agosto de 2020). Todos os participantes assinaram o Termo de Consentimento Livre e Esclarecido, assinado em duas vias, sendo uma entregue a pessoa idosa.

## Resultados

Em relagáo as variáveis sociodemográficas dos 49 participantes, houve predomínio do sexo feminino 81,60%(n=40), faixa etária de 60 - 79 anos 81,60%(n=40), com companheiro 53,10%(n=26), escolaridade entre 1 a 4 anos de estudo 57,10%(n=28), mora com companheiro 79,60%(n=39) e com renda média familiar de dois salários mínimos 38,80%(n=19) (Tabela 1). A idade média foi de 71,8 (DP= 8,72), com mínimo de 61 e máximo de 105 anos. A média do total de doengas de doengas 5,73 (DP=3,37).

Na segunda avaliagáo, a média da fragilidade 7,96 dobrou em comparagáo com a média da primeira 3,20. Em relagáo a AIVD, a média foi de A2 = 18,55, e a AVD, a média foi de A2 = 89,18, apresentando um comprometimento dos participantes em relagáo a realizagáo de suas atividades em comparagáo com a primeira avaliagáo (Tabela 2).

**Tabela 1:** Caracterizado sociodemográfica das pessoas idosas com Diabetes Mellitus no seguimento de dezoito meses (n=49). Ribeirao Preto, Sao Paulo, Brasil, 2019-2021

Categoria	%(n)
Sexo	
Feminino	81,60(40)
Masculino	18,40(9)
Idade	
Pessoa idosa mais jovem (60-79 anos)	81,60(40)
Pessoa idosa mais velha (>80 anos)	18,40(9)
Estado Civil	
Com companheiro	53,10(26)
Sem companheiro	46,90(23)
Escolaridade	
Analfabeto	2,10(1)
1 a 4 anos	57,10(28)
5 a 9 anos	26,50(13)
10 ou mais anos	14,30(7)
Mora sozinho	
Sim	20,40(10)
Nao	79,60(39)
Renda familiar SM (Salário-Mínimo)	
1 SM	26,50(13)
2 SM	38,80(19)
3 a 5 SM	28,60(14)
6 a 9 SM	6,10(3)

**Tabela 2:** Valor do índice de validade de conteúdo por itens individuais do conteúdo por juízes especialistas em aparencia. Fortaleza, CE, Brasil, 2018

Variáveis	Média	Mediana	Mínimo	Máximo	DP	^p^
Fragilidade						0,002
A1	3,20	3,00	0	8	2,245	
A2	7,96	7,00	0	14	2,715	
AIVD						0,023
A1	19,31	21,00	7,00	21,00	3,00	
A2	18,55	20,00	7,00	21,00	3,22	
AVD						<0,001
A1	94,39	100,00	30,00	100,00	12,48	
A2	89,18	95,00	20,00	100,00	16,44	

p - nivel de significancia: p < 0,05; DP: Desvio - Padreo; Teste estatístico: Wilcoxon pareado Atividades Instrumentais da Vida Diária (AIVD). Atividades Básicas da Vida Diária (AVD)

**Figura 1:**
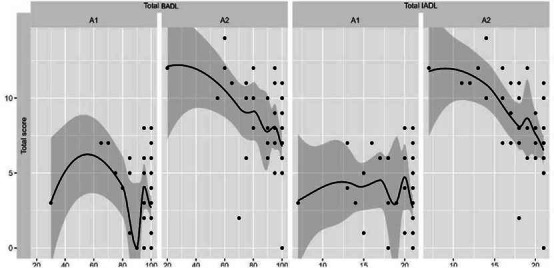
Evoluáo das AVD e AIVD das pessoas idosas com Diabetes Mellitus no seguimento de dezoito meses (n=49). Ribeiráo Preto, Sáo Paulo, Brasil, 2019-2021. Atividades Instrumentais da Vida Diária (AIVD). Atividades Básicas da Vida Diária (AVD)

No que tange as AVD e AIVD é possível observar gráficamente o comprometimento de tais atividades entre as avaliagóes A1 e A2 (Figura 1).

Na análise, utilizando-se regressao linear múltipla para o desfecho (escore total de fragilidade), ocorreu a perda ou piora da funcionalidade no início do seguimento (p=0,028) e o aumento do número total de doengas (p=0,035), sendo que impactaram negativamente sobre a fragilidade (Tabela 3). Ou seja, pessoas idosas com maior comprometimento funcional e maior número de morbidades, na primeira avaliagao (basal), apresentaram-se mais frágeis no período de seguimento (segunda avaliagao) subsequente.

**Tabela 3:** Fatores associados a fragilidade de pessoas idosas com Diabetes Mellitus no seguimento de dezoito meses (n=49). Ribeiráo Preto, Sáo Paulo, Brasil, 2019 - 2021

Variáveis	P	(IC 95%)	^p^
Sexo	0,04	(-2,138 - 1,591)	0,769
Idade	0,12	(-,053 - ,128)	0,406
Escore AIVD antes	-0,34	(-,0582 - -,036)	0,028
Total de doencas	0,30	(0,017 - 0,459)	0,035

p: Beta; p - nivel de significancia: p < 0,05; Teste estatístico: Regressao Linear Múltipla; IC: Intervalo de Confianza

## Discussao

A Atengáo Primária a Saúde atua como porta de entrada para os usuários do sistema de saúde por meio de agóes de promogáo, prevengáo, tratamento e reabilitagáo no manejo de condigóes que afetam a saúde da populagáo, com destaque para doengas crónicas. O Diabetes Mellitus é uma condigáo crónica que afeta principalmente a populagáo idosa, e o monitoramento e avaliagáo longitudinal deste público com a doenga podem influenciar diretamente o desfecho do quadro clínico, evitando complicagóes, hospitalizagóes e morte^4^^,^^16^.

A pandemia da Covid-19 trouxe mudangas significativas na vida da populagáo, especialmente, desse grupo populacional e, com efeito, sem precedentes em todo o mundo. O isolamento social, a solidáo e o aumento da dependencia afetaram diretamente a qualidade de vida destas pessoas, resultando no aparecimento de alguns problemas de saúde e síndromes geriátricas como a fragilidade^17^.

O aparecimento da fragilidade pode ser influenciado o aumento da idade e presenga de multimorbidades, dentre elas, o Diabetes Mellitus. Esta síndrome é caracterizada por uma incapacidade de o organismo lidar com os agentes estressores, elevando o risco de incapacidades. A ocorrencia concomitante da fragilidade e DM pode elevar as chances de complicagóes graves para a pessoa idosa, além de comprometer o desenvolvimento das AVD e AIVD^18^^,^^19^.

Neste estudo, a fragilidade, AVD e AIVD sofreram importantes alteragóes. Foi identificado que o processo de fragilizagáo aumentou e ocorreu diminuigáo nos níveis de independencia no período pandemico. Convém destacar que o aumento da fragilidade leva a diminuigáo das atividades cotidianas na populagáo idosa^20^^,^^21^. Esta redugáo pode estar atrelada ao estilo de vida, pois algumas pessoas idosas, antes da pandemia, conseguiam de forma independente realizar seus afazeres cotidianos, mas no cenário da Covid-19 tiveram que recorrer ao isolamento social para protegáo e exposigáo desnecessárias ao vírus, pois eram um dos grupos de maior risco para contaminagáo^22^. Ressalta-se que as AIVD sofreram maior comprometimento quando comparada as AVD, fato atrelado a impossibilidade de desenvolver algumas habilidades sociais e complexas. Contudo, ocorreu a diminuigáo de ambas devido ao processo confluencia e hierarquizagáo que interligam tais atividades^18^.

Destaca-se, ainda, neste estudo a relagáo entre fragilidade, número de doengas e AIVD, onde o processo de fragilizagáo foi afetado negativamente pelo aumento do comprometimento funcional e do número de morbidades, na qual a presenga da DM pode ter intensificado esse processo^23^^,^^24^, o que pode ter relagáo direta com o período de isolamento na pandemia que tornou a populagáo idosa ainda mais vulnerável a estressores e ao surgimento de doengas, assim como diminuiu sua prática de atividades físicas e sociais^25^.

Estudo realizado na regiáo Nordeste do Brasil em 2020, demonstrou que a manutengáo da capacidade funcional em pessoas idosas diabéticas pré-frágeis aumentou em 38,00% a chance de náo se tornarem frágeis e ressaltou que a presenga de outras comorbidades pode influenciar a alteragáo da funcionalidade e da fragilidade, corroborado pelo presente estudo4. Uma pesquisa realizada na regiáo Centro-Oeste do Brasil, em 2020, verificou uma associagáo entre o elevado número de doengas em pessoas idosas pré-frágeis e frágeis, assim como a associagáo de DM com o maior risco de fragilidade^26^.

Revisáo sistemática com metanálise, realizada com 32 estudos em 2021, trouxe evidencias de que pessoas idosas com DM possuem maior suscetibilidade a ter fragilidade do que aqueles sem a doenga, e que o comprometimento funcional pode ter influencia da DM e acelerar esse processo, além disso, a coexistencia com múltiplas doengas pode exacerbar os sintomas da fragilidade^27^.

Portanto, é premente o estabelecimento de um cuidado integral da populagáo idosa com doengas crónicas, com destaque para os que possuem DM, pois a presenta de tal morbidade possui uma relagáo intrínseca com o aparecimento da fragilidade e comprometimento de AVD e AIVD. A prestagáo de um acompanhamento longitudinal e contínuo por profissionais de saúde com foco no controle da DM e rastreio precoce da fragilidade e perda da capacidade funcional podem contribuir para promogáo de um envelhecimento ativo e saudável.

O estudo destaca como limitagáo a perda amostral entre as duas avaliagóes, possivelmente influenciada pelo período pandemico ocasionado pela Covid-19. Contudo, os resultados obtidos mostraram que a inatividade física e o isolamento social contribuíram para a evolugáo da fragilidade e o comprometimento da capacidade funcional.

## Conclusao

A Atengáo Primária a Saúde tem papel fundamental para o cuidado de pessoas idosas com Diabetes Mellitus, pois contribui para a identificagáo de condigóes que podem afetar a saúde deste grupo populacional.

A fragilidade é uma das síndromes mais prevalentes em pessoas idosas com o diagnóstico de DM. A evolugáo do aumento da fragilidade e a sua associagáo com fatores como comprometimento funcional de AVD, AIVD e o número de doengas identificados neste estudo impactaram nas condigóes de saúde e atividades cotidianas.

A investigagáo da fragilidade e da funcionalidade na populagáo idosa com doengas crónicas náo transmissíveis, com destaque para a DM, é premente para a garantia de um envelhecimento com qualidade de vida e ativo.

O estímulo para a implementagáo da educagáo permanente com os profissionais de saúde acerca do processo de fragilizagáo e capacidade funcional em pessoas idosas com DM pode contribuir para o desenvolvimento de intervengóes assertivas para prevengáo e/ou reversáo do nível de dependencia e, consequentemente, da fragilidade, sendo necessária sua incorporagáo permanente na rotina dos servigos de saúde, principalmente neste período pós- pandemia.
